# Reactivity against *Sarcocystis neurona* and *Sarcocystis falcatula*-like in horses from Southeastern and Midwestern Brazil

**DOI:** 10.1590/S1984-29612023031

**Published:** 2023-06-02

**Authors:** Thiago Merighi Vieira da Silva, Mariele De Santi, Luiz Ricardo Gonçalves, Márcia Mariza Jusi Merino, Marcos Rogério André, Rosangela Zacarias Machado

**Affiliations:** 1 Laboratório de Agentes Transmitidos por Vetores Artrópodes, Departamento de Patologia, Reprodução e Saúde Única, Faculdade de Ciências Agrárias e Veterinárias – FCAV, Universidade Estadual Paulista “Júlio de Mesquita Filho” – UNESP, Jaboticabal, SP, Brasil; 2 Imunodot Diagnósticos, Jaboticabal, SP, Brasil

**Keywords:** *Sarcocystis* spp., IFAT, EPM, horses, cross-reactivity, *Sarcocystis* spp., RIFI, MPE, equinos, reatividade-cruzada

## Abstract

Equine protozoal myeloencephalitis (EPM) is a neurological disease caused by *Sarcocystis neurona*. Immunofluorescence antibody tests (IFATs) have been widely used to identify exposure of horses to *S. neurona* in Brazil. Here we used IFAT to search for IgG antibodies against *Sarcocystis falcatula*-like (Dal-CG23) and *S. neurona* (SN138) in sera from 342 horses sampled in Campo Grande, Mato Grosso do Sul state (Midwestern), and São Paulo, São Paulo state (Southeastern), Brazil. The 1:25 cutoff value was chosen to maximize sensitivity of the test. IgG antibodies against *S. neurona* were detected in 239 horses (69.88%), whereas IgG antibodies against *S. falcatula*-like were detected in 177 horses (51.75%). Sera from 132 horses (38.59%) reacted against both isolates. Absence of reactivity was evidenced in 58/342 horses (16.95%). The lower cutoff used, and the presence of opossums infected with *S. falcatula*-like and *Sarcocystis* spp. in the regions where the horses were sampled, might justify the high seroprevalence observed here. Owing to the similarity among antigens targeted in immunoassays, reports on *S. neurona*-seropositive horses in Brazil may also derive from the exposure of horses to other *Sarcocystis* species. The role of other *Sarcocystis* species in causing neurological diseases in horses in Brazil remains unclear.

## Resumo

Mieloencefalite protozoária equina (MPE) é uma doença neurológica causada por *Sarcocystis neurona*. Reação de imunofluorescência indireta (RIFI) tem sido utilizada para identificar a exposição de equinos à *S. neurona* no Brasil. Neste estudo, a RIFI foi utilizada para avaliar a presença de anticorpos IgG anti-*Sarcocystis falcatula*-like (Dal-CG23) e anti-*S. neurona* (SN138) no soro de 342 equinos de Campo Grande, Mato Grosso do Sul (Centro-oeste) e São Paulo, São Paulo (Sudeste), Brasil. O ponto de corte de 1:25 foi escolhido para maximizar a sensibilidade. Anticorpos IgG anti-*S. neurona* foram detectados em 239 cavalos (69,88%), enquanto anticorpos IgG anti-*S. falcatula*-like foram detectados em 177 cavalos (51,75%). O soro de 132 animais (38,59%) reagiu contra ambos os isolados. A ausência de reatividade foi evidenciada em 58/342 animais (16,95%). O baixo ponto de corte e a presença de gambás infectados com *S. falcatula*-like e *Sarcocystis* spp., nas regiões onde os equinos foram amostrados podem justificar a alta soroprevalência aqui observada. Devido à similaridade entre antígenos de superfície detectados nos imunoensaios, relatos de soropositividade contra *S. neurona* no Brasil podem resultar da exposição dos equinos a outras espécies de *Sarcocystis*. O papel de outras espécies de *Sarcocystis* como causa de doença neurológica em equinos no Brasil permanece incerto.

## Introduction

Equine protozoal myeloencephalitis (EPM) is a neurological disease caused by *Sarcocystis neurona* (Dubey et al., 1991). Opossums (*Didelphis* spp.) are the definitive hosts and sources of *S. neurona* infections in horses. *Didelphis virginiana* is the definitive host of *S. neurona* in North America (Fenger et al., 1995), and *Didelphis albiventris* is the definitive host of *S. neurona* in Brazil (Dubey et al., 2001). Opossums are definitive hosts also for *Sarcocystis speeri, Sarcocystis lindsayi*, and *Sarcocystis falcatula.* Coinfection with different *Sarcocystis* species have already been described in opossums (Dubey et al., 2016).

In Brazil, the majority of *Sarcocystis* shed by opossums has been identified as *Sarcocystis falcatula*-like due to genetic characteristics and/or experimental infectivity to budgerigars (*Melopsittacus undulatus*) (Gondim et al., 2019; Acosta et al., 2018; Cesar et al., 2018). *Sarcocystis falcatula*-like in Brazil present high allelic variation in genes coding for immunodominant surface antigens (*SAGs*), which may represent genetic recombination between *S. neurona*, *S. falcatula*, or other *Sarcocystis* species shed by opossums (Monteiro et al., 2013; Cesar et al., 2018; Gondim et al., 2019).

Serological techniques such as immunofluorescence antibody tests (IFAT), Western blot (WB), and enzyme-linked immunosorbent assays (ELISAs) based on *SAGs* have been used to identify the exposure of horses to *S. neurona* in Brazil [summarized by Gondim et al. (2021)]. *Sarcocystis neurona* has also been evidenced in central nervous system of horses in Brazil by means of immunohistochemistry (Masri et al., 1992; Paixão et al., 2007; Henker et al., 2020) and molecular methods (Henker et al., 2020). However, *S. neurona* has never been isolated from horses in the country.

Due to the high similarity among *SAGs* in *Sarcocystis* shed by opossums, serological cross-reactivity between *S. neurona* and *S. falcatula-*like was hypothesized, being later demonstrated in experimentally infected Mongolian gerbils (*Meriones unguiculatus*) (Jesus et al., 2019). The aim of the present study was to evaluate horse sera reactivity against *S. falcatula*-like (strain Dal23-CG isolated from a *D. albiventris* from Campo Grande) and *S. neurona* (North American strain SN138) among horses sampled in the cities of Campo Grande, Mato Grosso do Sul state (Midwestern), and São Paulo, São Paulo state (Southeastern), Brazil.

## Material and Methods

### Samples

To detect anti-*S. neurona* and anti-*S. falcatula*-like antibodies, IFAT was performed using serum samples obtained by convenience from 342 horses. Samples from 262 horses were collected in 12 properties located in the peri-urban region of Campo Grande for screening of *Rickettsia*, *Borrelia*, and Anaplasmataceae agents, and were kindly supplied by Dr. Heitor Miraglia Herrera from the Universidade Católica Dom Bosco (UCDB). Samples from 30 horses were collected for routine exams and were kindly supplied by the veterinary staff of the military police cavalry from Campo Grande. Samples from 50 horses were collected for routine exams and were kindly supplied by the veterinary staff of an equestrian club in São Paulo. The samples included males and females of diverse ages and breeds (Supplementary Table S1).

### Merozoites and antigen production

Merozoites of *S. neurona* (strain SN138) isolated from *Didelphis virginiana* (Lindsay et al., 2004) and *S. falcatula*-like (strain Dal23-CG) isolated from *Didelphis albiventris* (De Santi et al., 2023), were used as antigens. The parasites were propagated in T25 flasks with confluent monolayers of Vero cells (BCRJ:0245), supplemented with Iscove’s Modified Dulbecco’s Medium (IMDM) (Invitrogen/Gibco^®^, Carlsbad, USA), 1% antibiotic–antimycotic (100 units/mL of penicillin, 100 μg/mL of streptomycin and 0.25 μg/mL of amphotericin B; Gibco^®^, Carlsbad, USA), and 5% inactivated fetal bovine serum (Invitrogen/Gibco^®^, Auckland, NZ). Flasks were maintained at 37 °C in a humidified incubator with 5% CO_2_ (Nuare NU-4750E, Plymouth, MN, USA).

### Indirect Fluorescent Antibody Test (IFAT)

**Figure 1:**
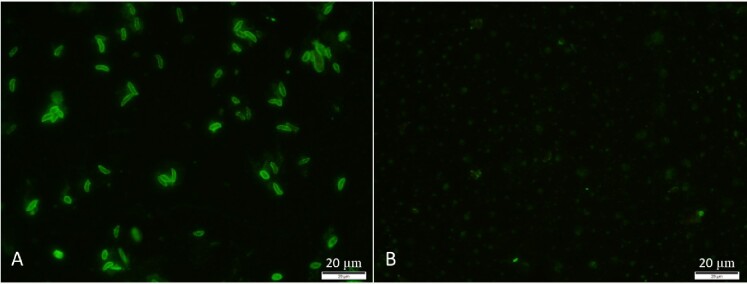
Indirect fluorescence antibody test (IFAT) in horses. *Sarcocystis falcatula*-like and *Sarcocystis neurona* were used as antigens. A- Positive titer with merozoites of *Sarcocystis neurona*. 400x. B- Negative control with absence of fluorescence. 400x.

Extracellular merozoites were harvested from the flasks, purified using a sterile 5 μm pore size filter, washed in phosphate-buffered saline (PBS) by centrifugation (1500 × g for 5 min), fixed in 10% formalin for 10 min, washed twice in PBS, and used to cover 12-well slides in 10 μL aliquots per well. The slides were allowed to air dry and stored at -20 °C. Prior to use, the slides were removed from storage and allowed to thaw at room temperature (approximately 22 °C) for 30 min. IFAT was performed as previously described (André et al., 2010). Briefly, serum samples were diluted at 1:25 in PBS and pipetted into each well. The slides were incubated in a humid chamber at 37 °C for 45 min, washed for 15 min with PBS, and dried at 37 °C. The delimited areas were covered with 10 μL of commercial fluorescein-labeled anti-horse immunoglobulin G (IgG) (Sigma–Aldrich^®^, St. Louis, USA) at a 1:64 dilution in PBS containing 10% Evans blue. The slides were then incubated for 45 min in a dark and humid chamber, washed, and dried. For microscopic evaluation, slides were overlaid with buffered glycerin (pH 8.7), covered with glass coverslips, and examined at 400 x magnification using an epifluorescence microscope (Olympus, Japan). Only samples presenting fluorescence in the entire periphery of merozoites were considered positive (Figure 1). Positive controls consisted of sera from horses that reacted at 1:100 solely for each parasite (*S. neurona* or *S. falcatula*–like). Negative controls consisted of horse sera that tested negative for both parasites at 1:25 dilution. Positive samples at 1:25 were further diluted in two-fold increments to obtain the final titer, with the endpoint titer being the last serum dilution showing distinct whole parasite fluorescence.

## Results and Discussion

From the 342 horse serums analyzed by IFAT in the present study, 194 were from male and 148 from female. A total of 14 different breeds were included in the study and are described in Supplementary Table 1. A total of 239 samples (69.88%) reacted against *S. neurona* (209 from Campo Grande and 30 from São Paulo), whereas 177 samples (51.75%) reacted against *S. falcatula*-like (163 from Campo Grande and 14 from São Paulo). IgG antibodies against both *S. neurona* and *S. falcatula*-like were detected in 132/342 samples (38.59%) (122 from Campo Grande and 10 from São Paulo). Absence of reactivity was evidenced in 58/342 samples (16.95%) (42 from Campo Grande and 16 from São Paulo) (Table 1). A total of 107 horses (31.28%) presented IgG antibodies solely against *S. neurona* (87 from Campo Grande and 20 from São Paulo), while 45 horses (13.15%) presented IgG antibodies solely against *S. falcatula*-like (41 from Campo Grande and 04 from São Paulo). Titers for *S. falcatula*-like and for *S. neurona* are described in Table 2.

**Table 1:** Seroprevalence for *Sarcocystis falcatula*-like and *Sarcocystis neurona* in 342 horses from Campo Grande Mato Grosso do Sul state (Midwestern), and São Paulo, São Paulo state (Southeastern) tested by IFAT.

		*Sarcocystis falcatula*-like			*Sarcocystis neurona*			*Sarcocystis neurona* and *Sarcocystis falcatula*-like	
Seropositive		51.75% (177/342)	Campo Grande = 163		69.89% (239/342)	Campo Grande = 209		38.59% (132/342)	Campo Grande = 122
			São Paulo = 14			São Paulo = 30			São Paulo = 10
Seronegative		48.25% (165/342)	Campo Grande = 129		30.11% (103/342)	Campo Grande = 83		16.95% (58/342)	Campo Grande = 42
			São Paulo = 36			São Paulo = 20			São Paulo = 16

**Table 2:** Titration of *Sarcocystis falcatula*-like and *Sarcocystis neurona*-seropositive horses from Campo Grande Mato Grosso do Sul state (Midwestern), and São Paulo, São Paulo state (Southeastern) tested by IFAT.

Titers	*Sarcocystis falcatula*-like			*Sarcocystis neurona*	
	Campo Grande	São Paulo		Campo Grande	São Paulo
1:25	69	1		49	20
1:50	38	2		84	3
1:100	39	6		47	1
1:200	13	5		20	3
1:400	4	0		9	2
1:800	0	0		0	1
Total	163	14		209	30

Results obtained by IFAT indicated that horse sera tested in our study reacted to more than one *Sarcocystis* species. Notably, 69.88% (239/342) of the samples reacted against *S. neurona*, and 51.75% (177/342) reacted against *S. falcatula*. This is a high seroprevalence compared to previous investigations conducted in Brazil. These include horse serum analyzed by IFAT in the states of Alagoas (Northeast, 2.8%) (Valença et al., 2019), Mato Grosso (Midwestern, 20.8%) (Borges et al., 2017), Minas Gerais (Southeastern, 23.09%) (Ribeiro et al., 2016), Rio Grande do Sul (South, 33.86%) (Antonello et al., 2015), São Paulo (Southeastern, 23.8%) (Oliveira et al., 2017) and Roraima (North, 40.4%) (Gomes et al., 2019). In the present study, the highest titer observed for *S. neurona* was 1:800, whereas 1:400 was the highest titer observed for *S. falcatula-*like. Titers up to 1:5120 were reported in 8.13% (7/86) of the *S. neurona*-seropositive horses in the state of Roraima (Gomes et al., 2019).

Discrepancies of the results presented in comparison with previous studies may be associated with the lower cutoff titer used here, and with the presence of opossums infected with *Sarcocystis falcatula*-like and *Sarcocystis* spp. in the same regions where the horses were sampled (De Santi et al., 2023). A starting serum dilution of 1:25 was selected based on previous study from Duarte et al. (2003), who observed that a gold standard panel of horses infected with *S. neurona* showed seropositivity starting at 1:20 at IFAT. The authors recommended a 1:80 cutoff in IFAT for horses suspected to have EPM. Although the clinical history of the horses in this study was not available, none of the animals presented neurological signs at the time of blood collection, therefore a less conservative cutoff was used. Exposure to different *Sarcocystis* species may also generate different immunological responses (Borges-Silva et al., 2020). This could explain why horses in the present study reacted differently to *S. neurona* and *S. falcatula*-like antigens, with up to 8-fold increase in titers for *S. falcatula-*like and up to 16-fold increase in titers for *S. neurona*. It is not clear if the exposure to a *Sarcocystis* spp. could protect the horse from EPM caused by *S. neurona*.

Serological assays are the main tools for *ante mortem* diagnosis of EPM. However, they only indicate whether horses have been exposed to *S. neurona*, not providing certainty of current infection (Dubey et al., 2015). Furthermore, surface antigens, targeted in serological tests, are highly similar among *Sarcocystis* spp. shed by opossums (Gondim et al., 2021). Therefore, immunological assays that use whole merozoites as antigens, such as IFAT, and SAG-based ELISAs, may result in the false assumption that horses are seropositive for *S. neurona* due to seroconversion after infection with other *Sarcocystis* species. Confirmation of IFAT using immunoblot assays revealed a low number of “true positive” samples in horse sera tested against *S. neurona* (SN37R) in the state of Alagoas (Valença et al., 2019). In an IFAT survey performed with serum samples from 189 mares, 57 from the 64 samples that reacted against *S neurona*, reacted also against *Sarcocystis cruzy*, i.e., only seven samples reacted solely against *S. neurona* (Antonello et al., 2015). The presence of specific antibodies in serum and cerebrospinal fluid, associated with typical clinical signs, and the complete differential diagnosis, is currently used to define EPM diagnosis (Dubey et al., 2015).

The hypothesis of cross reactivity between different *Sarcocystis* species shed by opossums in Brazil could justify the high seropositivity observed for *S. neurona* in horses, despite the low number of reports on EPM in the country (Gondim et al., 2021). Cross reactivity between *S. neurona* and *S. falcatula*-like has already been observed in Mongolian gerbils (*Meriones unguiculatus*) experimentally infected and tested by Western blot (Jesus et al., 2019). Interestingly, cross reactivity was not observed when the same animals were tested by IFAT. However, cross reactivity between *S. neurona* and *S. falcatula*-like has not been proven in horses to the date. It is worth to note that not all horses infected with *S. neurona* develop clinical disease. The exact mechanisms that favor an asymptomatic infection to progress to a severe neurological disease remains unclear.

Based on the results presented here along with that from previous studies, is clear that the high seroprevalence of *S. neurona* reported in horses in Brazil should be interpreted with caution. Further studies are necessary to elucidate the role of *Sarcocystis* species other than *S. neurona* in infecting and causing clinical diseases in horses in the country.

## Supplementary Material

Supplementary material accompanies this paper.

Supplementary Table S1 Seropositivity for *Sarcocystis falcatula*-like and *Sarcocystis neurona* in horses from Campo Grande, state of Mato Grosso do Sul (Midwestern), and São Paulo, state of São Paulo (Southeastern), Brazil, tested by IFAT.

This material is available as part of the online article from https://doi.org/10.1590/S1984-29612023031
