# Supplementary material for: Reactivity against Sarcocystis neurona and Sarcocystis falcatula-like in horses from Southeastern and Midwestern Brazil
Source: Rev Bras Parasitol Vet. 2023 Jun 2;32(3):e007623. doi: 10.1590/S1984-29612023031 (PMC10259416; doi:10.1590/S1984-29612023031)
Supplement: Supplementary Table S1 [file rbpv-32-3-e007623-Suppl.pdf]

Supplementary Table S1: Seropositivity for *Sarcocystis falcatula*-like and *Sarcocystis neurona* in 342 horses from Campo Grande, state of Mato Grosso do Sul (Midwestern), and São Paulo, state of São Paulo (Southeastern), Brazil, tested by IFAT.

	Sarcocystis falcatula-like	*Sarcocystis neurona*	Double positive	Negative	Total
Male	30	55	79	30	194
Female	15	52	53	28	148
Belgian Warmblood	-	1	1	-	2
Dutch Warmblood	1	2	-	2	5
Anglo-Arabian	-	-	2	1	3
American Quarter Horse	7	50	42	31	130
Brazilian Sport Horse	12	24	39	12	87
American Paint Horse	-	3	-	1	4
Arabian	-	1	1	1	3
Mixed breed	17	17	32	3	69
Appaloosa	-	-	-	1	1
Pantaneiro	-	1	-	-	1
Mangalarga Marchador	2	3	4	4	13
Crioulo	2	1	5	-	8
Thoroughbred	2	2	5	1	10
Lusitano	2	2	1	1	6
